# Multi-informant validity evidence for the SSIS SEL Brief Scales across six European countries

**DOI:** 10.3389/fpsyg.2022.928189

**Published:** 2022-08-02

**Authors:** Christopher J. Anthony, Stephen N. Elliott, Michayla Yost, Pui-Wa Lei, James C. DiPerna, Carmel Cefai, Liberato Camilleri, Paul A. Bartolo, Ilaria Grazzani, Veronica Ornaghi, Valeria Cavioni, Elisabetta Conte, Sanja Tatalović Vorkapić, Maria Poulou, Baiba Martinsone, Celeste Simões, Aurora Adina Colomeischi

**Affiliations:** ^1^School of Special Education, School Psychology, and Early Childhood Studies, University of Florida, Gainesville, FL, United States; ^2^Sanford School of Social and Family Dynamics, Arizona State University, Tempe, AZ, United States; ^3^Department of Educational Psychology, Counseling, and Special Education, Pennsylvania State University, University Park, PA, United States; ^4^Centre for Resilience and Socio-Emotional Health, University of Malta, Msida, Malta; ^5^Department of Human Sciences for Education, University of Milano-Bicocca, Milan, Italy; ^6^Faculty of Teacher Education, University of Rijeka, Rijeka, Croatia; ^7^Department of Educational Sciences and Early Childhood Education, University of Patras, Patras, Greece; ^8^Department of Psychology, University of Latvia, Riga, Latvia; ^9^Faculty of Human Kinetics, University of Lisbon, Lisbon, Portugal; ^10^Faculty of Sciences in Education, Stefan cel Mare University, Suceava, Romania

**Keywords:** SSIS SEL Brief Scales, social and emotional learning, international assessment, concurrent validity, predictive validity, PROMEHS

## Abstract

The SSIS SEL Brief Scales (SSIS SEL*b*) are multi-informant (teacher, parent, and student) measures that were developed to efficiently assess the SEL competencies of school-age youth in the United States. Recently, the SSIS SEL*b* was translated into multiple languages for use in a multi-site study across six European countries (Croatia, Greece, Italy, Latvia, Portugal, and Romania). The purpose of the current study was to examine concurrent and predictive evidence for the SEL Composite scores from the translated versions of the SSIS SEL*b* Scales. Results indicated that SSIS SEL*b* Composite scores demonstrated expected positive concurrent and predictive relationships with scores from the Connor-Davidson Resilience Scale (CD-RISC) and negative relationships with scores from the problem behavior scales of the Strengths and Difficulties Questionnaire (SDQ). Although there were a few exceptions, these patterns generally were consistent across informants (parents, teachers, and students) and samples providing initial validity evidence for the Composite score from the translated versions of the SSIS SEL*b* Scales. Limitations and future research directions are discussed.

## Introduction

Children who can build and maintain social relationships, manage their thoughts, feelings and behaviors, and make responsible decisions are better prepared for success in school and life (e.g., Caprara et al., 2000; DiPerna et al., 2015, 2016, 2018; Mahoney et al., 2021). Yet, these Social Emotional Learning (SEL) skills do not automatically and ubiquitously develop in all children. For many children, specific school-based supports can be a key factor promoting the acquisition of these skills (e.g., Durlak et al., 2011). Awareness of these conclusions is rising across the globe, especially in the context of the social difficulties posed by the COVID-19 pandemic (Yoder et al., 2020; Grazzani et al., 2022). In the European context, increased attention and resources have been devoted to SEL and SEL programming for several years (e.g., Cefai et al., 2018a,b). Yet, implementation of SEL programming requires assessments that are thoughtfully developed, adapted for their specific intended application (e.g., screening), and validated for the interpretations and uses for which they are intended (Cavioni et al., 2020). Considering the linguistic diversity present in Europe, many countries simply do not have access to validated translations of high-quality SEL measures.

The purpose of the study was to address this need for high-quality SEL assessments by evaluating evidence concerning the validity of students’ scores on the SSIS SEL Brief Scales (SSIS SEL*b*; Elliott et al., 2020) as rated by parents, teachers, and students from six European countries. These students had participated in a multi-country, school-based mental health project (titled PROMEHS) where SEL competency was a key outcome variable (Cefai et al., in press). Specifically, we evaluated the concurrent and predictive validity of scores from the SSIS SEL*b* Scales (Elliott et al., 2020) by correlating them with scores from the Strengths and Difficulties Questionnaire (SDQ; Goodman, 1997), a measure of mental health, and the Connor-Davidson Resilience Scale (CD-RISC; Connor and Davidson, 2003), an adolescent self-report measure of resilience.

## European measurement of social emotional learning

Although comprehensive programs have been developed to promote SEL across schools in Europe, there is a lack of assessment compendia that recommend instruments to measure SEL, due in part to the scarcity of available measures intended for use across multiple countries. Cefai et al. (2021) recommended that SEL assessments be multi-modal, inclusive, and strengths-based to accurately assess students’ social and emotional skills and competencies. However, schools generally rely on vague guidelines for choosing an assessment, rather than a specified instrument catalog, resulting in less standardized and psychometrically valid assessment practices.

Recent projects have made promising strides toward developing comprehensive SEL assessments designed for use across European countries. For example, the European Assessment Protocol for Children’s SEL Skills developed and validated a SEL measure, *How One Feels (HOF)*, designed for use with children aged 6 through 10. This assessment consists of 10 vignettes that are designed to capture how the student predicts the character in the vignette will feel, and subsequently, how they will act (Cefai et al., 2021). Despite these recent efforts, most schools across Europe rely on translated versions of existing screeners with little established validity evidence, teacher observations, and various student self-report instruments to assess students’ SEL competencies.

The Promoting Mental Health at Schools (PROMEHS)^1^ project was in fact developed to address some of these issues on the promotion of mental health and social and emotional learning. In view of the lack of European based programs in SEL and mental health, the project aimed to design, implement and evaluate a mental health promotion curriculum in schools for students, school staff and parents, leading to the development of an evidence based, universal program for schools in Europe. This entailed the use of common measures which could be used across the six European program countries in evaluating the impact of the program and how this may vary across contexts. The SSIS-SEL Brief Scales were selected not only because they were sufficiently brief and user friendly but also they evaluate SEL as construed in the project, that is, the five domains in the CASEL SEL framework: self-awareness, self-management, social awareness, relationship skills, and responsible decision-making (Collaborative for Academic, Social, and Emotional Learning [CASEL], 2021).

### The SSIS SEL brief scales and the PROMEHS study

This study focuses on international validation of scores from the SSIS SEL Brief Scales (SSIS SEL*b*; Elliott et al., 2020), which are brief, multi-informant (teacher, parent, student) rating scales targeting children’s SEL. The SSIS SEL*b* were created using procedures grounded in Item Response Theory (IRT: Hambleton et al., 1991) to identify items that were most efficient for screening children likely struggling with SEL skills. Using the item pools from the full SSIS SEL (Gresham and Elliott, 2017), the SSIS SEL*b* authors developed 20-item rating forms for teachers (Anthony et al., 2021); parents (Elliott et al., 2021); and students (Anthony et al., 2020a). All forms were aligned with the prominent framework suggested by the (Collaborative for Academic, Social, and Emotional Learning [CASEL], 2021) that includes five interrelated domains: Self-Awareness; Self-Management; Relationship Skills, Social Awareness, and Responsible Decision-Making. Although subscale scores are available for each of these domains, the SSIS SEL*b* authors recommend that interpretation be focused on the SSIS SEL*b* Composite—an aggregate score representing each of the five CASEL domains equally. Previous studies support the reliability and validity of the SSIS SEL*b* scores (e.g., Elliott et al., 2020; Anthony et al., 2021) for students in U.S. schools.

The data featured in this report are part of the PROMEHS project completed by a team of European researchers in Croatia, Greece, Italy, Latvia, Portugal, and Romania. These investigators advanced the understanding of children’s social emotional learning (SEL) competencies as viewed by parents, teachers, and students themselves. To accomplish this work, the research team, in collaboration with the authors of the SSIS SEL Brief Scales, first translated and then tested the measurement invariance of the translated versions of SSIS SEL*b* assessments with parents’, teachers’, and students’ representative of their respective countries (Anthony et al., Accepted). Specifically, the measurement invariance of the translated SSIS SEL*b* versions was examined with data from a sample of 10,609 teacher ratings, 8,549 parent ratings, and 6,611, student ratings in 2020–2021. Results revealed a high degree of measurement invariance, supporting the use of the SSIS SEL*b* for comparative research across these countries. Further evidence of the validity of these same scores, along with other concurrent and translated measures of the students’ social emotional functioning are needed before making confident conclusions about the effects of the PROMEHS project on students’ social emotional competence. When providing validity evidence for scores from a measure, it is also important to consider the strength of validity evidence for criterion measures. Unexpected or disappointing results from validity analyses can indicate problems with either the measure under investigation or the outcome measures (or both) and thus using more evidence-based outcome measures increases the likelihood of generating meaningful validity data. The outcome measures chosen for this study were the Strengths and Difficulties Questionnaire (SDQ; Goodman, 1997) and the Connor-Davidson Resilience Scale (CD-RISC; Connor and Davidson, 2003). The SDQ measures the mental health of school children as reported by teachers, parents, and students themselves. This measure includes four negatively valenced subscales (Emotional Symptoms; Conduct Problems; Hyperactivity/Inattention; Peer Relationship Problems) and one positively valenced subscale (Prosocial Behavior). The CD-RISC is a self-report measure of children’s resilience, a construct that has been linked with SEL skills (Reyes et al., 2013). There is extensive evidence supporting the reliability and validity of scores from these measures overall including a relatively large amount of evidence specifically gathered in most of the countries included in this project. For examples of prior psychometric research on the SDQ and CD-RISC in these countries, see Table 1 and for an explication of this evidence, see Supplementary Material.

**TABLE 1:** Summary of psychometric studies of validity measures across countries.

	Teacher	Parent	Student/Self report
**Croatia**			
SDQ	Cronbach α’s from 0.63 to 0.84 across subscales; Five-factor structure explaining 57.35% of total variance (Tatalović Vorkapič et al., 2017)	None	CFI equal to 0.84 and RMSEA equal to 0.05 for five-factor model (Stevanovic et al., 2015)
CD-RISC	–	–	Resilience was negatively correlated (*r* = -0.25) with a Maternity Blues scale (Mikuš et al., 2020)
**Greece**			
SDQ	Cronbach α’s from 0.81 to 0.92 across subscales; Factor loadings for the five-factor model ranging from 0.42 to 0.81 (Bibou-Nakou et al., 2019)	Cronbach α for total difficulties score equal to 0.78; Moderate to high correlations between parent SDQ and KIDSCREEN-52 (Giannakopoulos et al., 2013)	Cronbach α for total difficulties score equal to 0.77; Moderate to high correlations between self-report SDQ and KIDSCREEN-52 (Giannakopoulos et al., 2013)
CD-RISC	–	–	Cronbach α of 0.93; Resilience scores significantly negatively correlated (−0.67) with Perceived Stress Scale (Tsigkaropoulou et al., 2018)
**Italy**			
SDQ	Cronbach α’s from 0.56 (conduct problems) to 0.81 (total difficulties score); CFA for five-factor model showed RMSEA = 0.048 (Tobia and Marzocchi, 2018)	Cronbach α’s from 0.56 (conduct problems) to 0.81 (total difficulties score); CFA for five-factor model showed RMSEA = 0.048 (Tobia and Marzocchi, 2018)	Cronbach α’s from 0.66 to 0.72 for total difficulties scale and from 0.64 to 0.70 for prosocial behavior; All factor loadings significant for a three-factor model (Di Riso et al., 2010)
CD-RISC	–	–	Cronbach α equal to 0.84; Internalizing and externalizing problems reported to be negatively and significantly correlated with resilience (Grazzani et al., 2022)
**Latvia**			
SDQ	Cronbach α equal to 0.70 across subscales; (Martinsone et al., 2022)	Spearman’s r correlation between Autism Spectrum Quotient-10-Child (AQ-10) and SDQ equal to 0.67; Parent SDQ found to be highly correlated with Signposting Questionnaire for Autism, for the whole sample (Jones et al., 2020)	None
CD-RISC	–	–	None
**Portugal**			
SDQ	Cronbach α equal to 0.80 for the prosocial behavior scale and two positively worded items from the Peer Problems subscale (Veiga et al., 2017)	Cronbach α’s ranging from 0.56 to 0.79; Acceptable fit with five-factor model with all items significantly loading onto their subscales (Costa et al., 2020)	Cronbach α’s ranging from 0.57 to 0.70; Concurrent validity demonstrated between the SDQ a related measure, the Youth Self-Report (Rodrigues et al., 2019)
CD-RISC	–	–	Evidence of concurrent validity with validated measures of stress, life satisfaction, mental health and physical health (Anjos et al., 2019)
**Romania**			
SDQ	None	None	Cronbach α’s ranging from 0.42 to 0.79 across all four difficulties subscales; No tested models demonstrated acceptable fit with the data, but the five-factor model performed the best comparatively (Sharratt et al., 2014)
CD-RISC	–	–	Cronbach α equal to 0.91; Results from a CFA supported a bidimensional model (Giurcă et al., 2021)

SDQ, Strengths and Difficulties Questionnaire; CD-RISC, Connor Davidson Resilience Scale.

### Research strategy and expected outcomes

Our research strategy was to explore validity evidence for the SSIS SEL*b* translations *via* examination of concurrent and predictive validity correlations between the SSIS SEL*b* SEL Composite and scores from the SDQ and CD-RISC across participating countries. We expected several broad outcomes to emerge:

1) Because they measure similar positive social behaviors, we expected scores from the SDQ Prosocial Behavior subscale to correlate positively and moderately with scores from the SSIS SEL*b* SEL Composite for all informants.
2) Based on prior literature establishing negative relationships between positive SEL skills and emotional behavior concerns (e.g., Elliott et al., in press; Giannakopoulos et al., 2013), we anticipated that scores from negatively valenced SDQ subscales (Emotional Symptoms; Conduct Problems; Hyperactivity/Inattention; Peer Relationships Problems) to correlate negatively and moderately to strongly with the SSIS SEL*b* SEL Composite Scores across informants.
3) Based on established relationships between SEL and resilience (e.g., Reyes et al., 2013), we expected scores from the CD-RISC, rated only by older students, to correlate moderately positively with the SSIS SEL*b* SEL Composite scores.

These anticipated outcomes specifically are in reference to concurrent validity correlations—we expected predictive validity correlations to be weaker in magnitude relative to their concurrent validity counterparts. Beyond evaluating these overall relationships, we compared them across countries to determine whether validity evidence was substantially different for any SSIS SEL*b* translation.

## Method

### Participants

Several samples were used to complete our analyses including a sample of teachers who completed the SSIS SEL*b*-Teacher SSIS SEL*b*-T and the SDQ; a sample of parents who completed the SSIS SEL*b*-Parent (SSIS SEL*b*-P) and the SDQ; and a sample of children who completed the SSIS SEL*b*-Student (SSIS SEL*b*-S), SDQ, and the CD-RISC (Table 2). These samples were gathered as part of a larger project evaluating the effects of a comprehensive mental health promotion intervention in schools. Two different samples were drawn for each informant: a concurrent validity sample consisting of both experimental and control cases at pretest, and a predictive validity sample consisting of only control cases who had data at both pretest and posttest. The broader samples came from 240 schools across the six countries (Croatia, Greece, Italy, Latvia, Portugal, and Romania) included in this study. Where possible, systematic sampling (e.g., sample every third student from the class roster), gender was balanced, and students from disadvantaged backgrounds (e.g., students with educational needs; migrant children) were included in the sample.^2^ Approximately 15% of children were reported by their teachers to belong to a disadvantaged group.

**TABLE 2:** Demographic characteristics of concurrent validity samples.

Characteristic	Greece			Croatia			Italy			Latvia			Portugal			Romania		
						
	T	P	S	T	P	S	T	P	S	T	P	S	T	P	S	T	P	S
**Concurrent validity sample**																		
*n*	1,600	732	206	1,112	853	128	2,256	1,382	749	1,792	1,636	732	1,677	1,326	836	1,772	2,087	979
Female	49.4	–	52.4	47.2	–	60.2	51.1	–	53.3	50.5	–	54.3	51.8	–	52.3	52.7	–	63.7
Grade																		
Kindergarten	39.1	–	–	37.8	–	–	30.9	–	–	29.2	–	–	20.5	–	–	33.9	–	–
Primary	39.4	–	–	31.8	–	–	32.1	–	–	36.9	–	–	34.0	–	–	26.6	–	–
Secondary	21.5	–	100.0	30.4	–	100.0	36.9	–	100.0	33.7	–	100.0	45.5	–	100.0	39.4	–	100.0
**Predictive validity sample**																		
*n*	356	105	47	386	242	51	589	256	148	922	783	353	538	391	227	497	427	55
Female	51.4	–	40.4	46.4	–	52.9	49.9	–	52.0	50.1	–	53.8	52.2		55.5	54.1	–	58.2
Grade																		
Kindergarten	56.5	–	–	38.9	–	–	36.0	–	–	30.3	–	–	17.5		–	40.2	–	–
Primary	30.6	–	–	24.1	–	–	29.7	–	–	38.5	–	–	32.7		–	21.9	–	–
Secondary	12.9	–	100.0	37.0	–	100.0	34.2	–	100.0	30.9	–	100.0	49.8		100.0	37.9	–	100.0

T, Teacher; P, Parent; S, Student.

Sample sizes reported post listwise deletion, so they reflect the demographics of samples used for analyses. Sample size for predictive validity sample reflects deletion of cases lost to attrition and non-control group participants. Some percentages do not sum to 100 due to rounding error and missing data.

Different demographic characteristics were available for each sample based on what information was deemed relevant for each informant. A full breakdown of demographic characteristics of participants can be found in Table 2.

### Measures

The focal measure of this study was the SSIS SEL*b*. Validity measures included the SDQ and the CD-RISC. In-depth information about the translations of these measures was discussed previously, but a brief explication of key psychometrics of the English versions of these measures follows. Reliability statistics consisting of Cronbach’s α calculated with the concurrent validity sample and test-retest reliability statistics calculated with participants from the control group who also had post-test data are presented in Table 3. Additionally, example items for each scale can be found in Supplementary Table 1.

**TABLE 3:** Reliability coefficients (Cronbach’s α) for analytic measures across countries.

Scale	Croatia		Greece		Italy		Latvia		Portugal		Romania	
						
	α	Test-retest	α	Test-retest	α	Test-retest	α	Test-retest	α	Test-retest	α	Test-retest
**Teacher**												
SSIS SEL*b* SEL composite	0.95	0.72	0.95	0.75	0.94	0.77	0.94	0.72	0.95	0.76	0.96	0.78
SDQ—Emotional symptoms	0.80	0.61	0.76	0.65	0.77	0.58	0.76	0.59	0.77	0.58	0.78	0.63
SDQ—Conduct problems	0.68	0.68	0.67	0.65	0.72	0.69	0.72	0.70	0.67	0.68	0.74	0.65
SDQ—Hyperactivity/inattention	0.85	0.82	0.84	0.81	0.84	0.80	0.86	0.78	0.85	0.79	0.84	0.74
SDQ—Peer relationship problems	0.69	0.68	0.61	0.59	0.65	0.59	0.67	0.62	0.61	0.63	0.59	0.58
SDQ—Prosocial	0.86	0.66	0.86	0.66	0.81	0.65	0.82	0.60	0.85	0.68	0.84	0.65
**Parent**												
SSIS SEL*b* SEL composite	0.88	0.68	0.87	0.37	0.85	0.64	0.90	0.68	0.88	0.56	0.91	0.56
SDQ—Emotional symptoms	0.63	0.55	0.64	0.45	0.66	0.60	0.66	0.61	0.64	0.60	0.72	0.57
SDQ—Conduct problems	0.38	0.48	0.50	0.40	0.57	0.52	0.54	0.61	0.52	0.59	0.63	0.47
SDQ—Hyperactivity/inattention	0.75	0.73	0.55	0.53	0.71	0.64	0.78	0.72	0.76	0.70	0.71	0.63
SDQ—Peer relationship problems	0.56	0.67	0.46	0.41	0.56	0.64	0.52	0.58	0.50	0.61	0.46	0.52
SDQ—Prosocial	0.66	0.66	0.66	0.45	0.63	0.68	0.66	0.59	0.65	0.55	0.69	0.54
**Student**												
SSIS SEL*b* SEL composite	0.82	0.61	0.84	0.07^ns^	0.83	0.52	0.84	0.60	0.87	0.56	0.86	0.46
SDQ—Emotional symptoms	0.74	0.28	0.75	0.23^ns^	0.76	0.75	0.74	0.60	0.66	0.57	0.77	0.41
SDQ—Conduct problems	0.41	0.30	0.39	0.11^ns^	0.54	0.54	0.40	0.46	0.53	0.55	0.52	0.32
SDQ—Hyperactivity/inattention	0.68	0.39	0.72	0.24^ns^	0.69	0.68	0.67	0.54	0.69	0.62	0.65	0.43
SDQ—Peer relationship problems	0.57	0.50	0.60	0.14^ns^	0.59	0.65	0.55	0.58	0.44	0.53	0.49	0.19^ns^
SDQ—Prosocial	0.66	0.67	0.54	-0.18^ns^	0.64	0.58	0.64	0.53	0.67	0.42	0.62	0.44
CD—Resilience	0.78	0.57	0.82	0.10^ns^	0.84	0.69	0.83	0.64	0.82	0.54	0.84	0.56

SDQ, Strengths and Difficulties Questionnaire; CD, Connor Davidson Resilience Scale. Unless otherwise noted, all coefficients were statistically significant (p < 0.05).

ns, not significant.

#### SSIS SEL Brief Scales

The SSIS SEL Brief Scales (SSIS SEL*b*) is a multi-informant assessment that evaluates the social-emotional skills of children and adolescents. The brief version of this assessment was developed from the SSIS SEL Rating Forms (Gresham and Elliott, 2017), and it is typically administered as a universal screening assessment that can be completed in less than 5 min. The items in this assessment were created to align with the CASEL framework for social-emotional learning competency (Anthony et al., 2020a). Three forms of the SSIS SEL*b* have been developed: the SSIS SEL*b* Teacher K-12 Form (SSIS SEL*b*-T), the SSIS SEL*b* Parent K-12 Form (SSIS SEL*b*-P), and the SSIS SEL*b* Student form (SSIS SEL*b*-S). Studies with representative samples of children from the U.S. indicate the scores from this assessment to be highly reliable and valid (e.g., Anthony et al., 2020a,b, 2021, 2022; Elliott et al., 2020). Specifically, alpha values for SEL composite scores were found to be 0.95 for teachers, 0.91 for parents, and 0.94 for students. High levels of concurrent validity also were evident when comparing scores from the SSIS SEL*b* and related measures, such as the Behavior Assessment System for Children—Second Edition (BASC-2; Reynolds and Kamphaus, 2004).

In the present study, the SSIS SEL*b* teacher, parent, and student versions were all translated for use in the six participating countries. Previous research has supported the use of the SSIS SEL*b* across various European countries, finding good levels of measurement invariance across translated versions (Anthony et al., Accepted). For the current samples, score reliability (Cronbach’s α and test-retest reliability coefficients calculated with the control sample) was generally strong with the notable exception of test-retest reliability coefficients for the Greek SSIS SEL*b*-S (Table 3).

#### Strengths and difficulties questionnaire

The SDQ is an emotional and behavioral screening questionnaire developed to measure the mental health of children and adolescents from 3 to 16 years old. This measure is comprised of five subscales, four of which assess difficulties (Emotional Symptoms, Conduct Problems, Hyperactivity/Inattention, Peer Relationships Problems), and one assesses strengths (Prosocial Behavior; Goodman, 1997). The SDQ consists of 25 items, each distributed evenly across the five subscales. All responses are rated on a 3-point Likert scale (0: “*not true*”; 1: “*somewhat true*”; 2: “*certainly true*”). The responses for the Prosocial Behavior subscale are reversed in valence so that the total score indicates the overall level of severity of problem behaviors.

Parent and teacher forms of the SDQ also are available, each consisting of the same 25 items found in the self-report form (Goodman, 1997). The psychometric properties of the SDQ have been validated in numerous studies in which the translated versions were used across the six European countries in the present study (Table 1). Translated versions of the self-report form of the SDQ have shown adequate levels of reliability and validity when implemented with Greek, Italian, and Romanian youth (Di Riso et al., 2010; Giannakopoulos et al., 2013; Sharratt et al., 2014). Further, the translated parent forms have also been validated by previous studies with Latvian and Portuguese participants (Costa et al., 2020; Jones et al., 2020). A sample of Croatian teachers completed the translated version of the SDQ teacher form, and results revealed moderate to high levels of reliability, supporting the use of the Croatian translation of the SDQ (Tatalović Vorkapič et al., 2017). For the current sample, reliability was generally strong across all informants (Table 3). Some exceptions to this rule for internal consistency included the Conduct Problems and Peer Relationship Problems scales of the parent and student report forms. With regard to test-retest reliability, the Greek self-report sample had notably lower test-retest reliability coefficients relative to other countries, as did, to a lesser extent, the Romanian student self-report sample and the Greek parent report sample.

#### Connor-Davidson Resilience Scale

The Connor-Davidson Resilience Scale (CD-RISC) is a self-report assessment used to measure resilience. For this study, we used a short form (Campbell-Sills and Stein, 2007) of the CD-RISC consisting of 10 items rated on a 5-point Likert-type scale with response options rated from 0 (“*not true at all*”) to 4 (“*almost always true*”). This measure has demonstrated good reliability and validity within various samples, and the original standardization studies yielded a five-factor structure (Connor and Davidson, 2003). Extant translations of the CD-RISC in the languages spoken by the six participating countries were administered for the present study where available. In countries where a translated CD-RISC had not been developed, such as Latvia, a translated version was created using a forward-backward translation process. Researchers consistently have reported translations of the CD-RISC are psychometrically valid in Croatia, Greece, Italy, Portugal, and Romania (e.g., Tsigkaropoulou et al., 2018; Anjos et al., 2019; Mikuš et al., 2020; Giurcă et al., 2021; Grazzani et al., 2022).

Reliability coefficients for the current sample were fairly strong with the exception of the test-retest reliability coefficient for the Greek sample.

### Procedures

In the piloting phase it was decided that 1,000 students were to be selected from each of the six participating country, clustered by group (experimental control) and by age (4–6, 8–10, 11–13, 15–16) to represent kindergarten, primary, lower secondary and upper secondary students. The 250 students selected from each age cohort were to be allocated equally in the experimental (125) and control (125) groups. The expected total sample size (6,000) would guarantee a maximum margin of error of 1.27% assuming a 95% confidence level.

Cluster sampling was used to select the schools ensuring good geographical representation (and age and school level), while stratified sampling was used to select the students from several classrooms within the selected schools. Selected students and their respective teachers and parents then completed the respective questionnaires. The administration of questionnaires was either completed online (in the case of teachers and older students) whilst in most instances parents completed the questionnaires manually and returned them sealed to the school; in such instances the researchers from that particular country then inputted all answers in the electronic data base. The data file was accessed only by the project evaluation team led by the University of Malta. Similarly, the primary school students completed the questionnaire manually in class, with data inputted into the electronic data base by the respective research teams. Ethical approval was obtained from the respective academic institutions and educational authorities and all participants gave their consent before completing the questionnaires.

The project evaluation team at the University of Malta in collaboration with the project coordination team at the University of Milano-Bicocca worked with the main researchers in the six implementing countries to ensure quality implementation and evaluation of the program. Implementation and evaluation guidelines, including translation and use of instruments, sample size, teacher training, and duration and frequency of implementation were agreed upon by the whole team to ensure consistency in program implementation and evaluation. In each trial country, a training support team was set to coordinate activities related to the training courses and supervisions of teachers, the translation and adaptation of the handbooks and guidelines and to organize and lead the meetings for school leaders and parents. Teachers in the experimental condition received 16 h of initial training in order to receive practical and theoretical knowledge about mental health promotion in the school context as well as tools and materials to implement the program. The training was carried out face to face and/or remotely depending on national COVID-19 health regulations. During the implementation, which lasted over a period of 6 months, teachers also received 9 h of mentoring and monitoring by qualified program trainers. The implementing teachers were provided with a manual of activities developed by the consortium as part of the project; their students and parents also received a handbook. A set of procedures were also applied to monitor the quality of the implementation across schools and countries. These included the assessment of program’s fidelity (the extent to which the implemented intervention corresponds to the originally intended program), dosage (which refers to how much of the intervention has been delivered), quality (related to how well different program components have been conducted), participants’ responsiveness (referring to the degree to which the program stimulates the interest and engagement of participants namely teachers, students, and parents) and adaptation (related to changes made in the original program during implementation.

Due to the COVID-19 situation, however, not all teachers were able to do 12 activities, with the number of sessions varying considerably particularly between countries due to health policies in place related to the pandemic. The majority of the 423 implementing teachers (59%) completed 10 or more activities, but 31% completed only 4 or fewer activities.

### Data analysis

Full sample sizes and demographics for participants can be found in Table 2. We used listwise deletion to handle all missing data because we had relatively small levels of incomplete cases and we aimed to evaluate the validity of scores from the SSIS and outcome measures as they are intended to be used (i.e., without imputation or proration). Missing data percentages were 5.1%, 5.2%, and 6.8% of all cases for concurrent validity testing of the SSIS SEL*b* Teacher, Parent, and Student forms, respectively. For the predictive validity testing, we excluded cases that were either lost to the study due to attrition or made coding errors on the identification variable that precluded determination regarding whether they had been in the treatment or control groups. For the Teacher, Parent, and Student surveys, 8.5%, 18.6%, and 21.4% of cases were lost to attrition from pre-test to posttest respectively and a further 15.2%, 23.7%, and 26.2% of cases were lost from pre-test to post-test due to coding errors. Of the remaining cases, we used control cases resulting in sample sizes of 3,288; 2,204; and 881 for the Teacher, Parent, and Student surveys respectively. Once accounting for these cases, there were no further missing data. Finally, the magnitude of all correlations was evaluated *via* Cohen’s (1988) criteria (i.e., small correlations ≈0.10; medium correlations ≈0.30; large correlations ≈0.50).

After addressing missing data, analyses were completed systematically to address the research questions. Our primary analysis consisted of a repeated set of correlational analyses in which the correlations between the SSIS SEL Brief Scales SEL Composite and our outcome measures (SDQ subscales and CD-RISC total score depending on SSIS SEL*b* form) were estimated. To evaluate statistically whether these correlations differed across countries, we estimated these correlations using multi-group structural equation modeling procedures. Specifically, we compared a model in which correlations were estimated freely for each of the six countries and compared this model with several models to statistically test differences in the magnitude of correlations across countries. We first conducted an omnibus test in which the freely estimated model was compared with a model in which all correlations were constrained to equality across country. If this test was statistically significant, we completed 16 model comparisons in which the freely estimated model was compared with a series of model testing individual pairwise comparisons between country (e.g., a model in which the correlation was held equal across Romania and Greece, but not across other countries). See Supplementary Figure 1 for a diagram of the model that enabled these analyses.

All analyses were completed using MPlus version 8.3 (Muthén and Muthén, 1998–2019) and the robust maximum likelihood estimator (MLR) to account for potential non-normality. All variables initially were standardized to ensure that raw covariances (which are manipulated in multi-group SEM models) were equivalent to correlations for model comparison purposes. Model comparisons were conducted using the Satorra-Bentler Chi Squared comparison approach (Satorra and Bentler, 2001) and multiple comparisons were accounted for using the Benjamini Hochberg false-discovery rate procedure (Benjamini and Hochberg, 1995). We completed our correlational analyses twice: first with concurrent data and second with predictive data. For concurrent correlations, we used pre-test data from the broader study and included both experimental and control cases because neither group had received intervention at pre-test. For predictive validity analyses, we only used control cases to ensure that the provision of the intervention being tested would not influence our results. All analyses were completed for teacher, parent, and student versions of the SSIS SEL*b* and outcome measures; however, the CD-RISC was only used with student respondents.

## Results

Mean scores and their standard deviations for all measures by country-focused samples and informants are documented in Table 4 (Concurrent validity sample) and Table 5 (Predictive validity sample). Validity correlations are presented in Table 6 and cross-country comparisons in validity correlations are found in Table 7.

**TABLE 4:** Means and standard deviations for concurrent validity samples across country.

Scale	Croatia	Greece	Italy	Latvia	Portugal	Romania
**Teacher**						
SSIS SEL*b* SEL composite	41.77 (10.69)	42.85 (10.53)	39.14 (11.14)	41.47 (10.32)	42.98 (10.53)	42.17 (11.71)
SDQ—Emotional symptoms	1.43 (2.01)	1.49 (1.91)	1.82 (2.13)	1.89 (1.99)	2.35 (2.23)	2.03 (2.08)
SDQ—Conduct problems	0.99 (1.46)	0.97 (1.44)	1.27 (1.73)	1.35 (1.73)	1.41 (1.72)	1.13 (1.62)
SDQ—Hyperactivity/inattention	2.91 (2.68)	2.71 (2.57)	2.86 (2.65)	3.32 (2.78)	3.54 (2.86)	2.61 (2.48)
SDQ—Peer relationship problems	1.62 (1.85)	1.53 (1.68)	1.79 (1.87)	2.11 (1.93)	1.65 (1.74)	2.08 (1.68)
SDQ—Prosocial	7.62 (2.33)	7.21 (2.43)	6.87 (2.41)	6.89 (2.31)	7.59 (2.32)	7.68 (2.19)
**Parent**						
SSIS SEL*b* SEL composite	42.53 (7.57)	44.47 (7.45)	42.33 (7.53)	37.06 (8.79)	43.98 (7.59)	44.43 (8.72)
SDQ—Emotional symptoms	1.25 (1.56)	1.93 (1.86)	2.09 (1.87)	2.24 (1.93)	2.81 (2.03)	2.78 (2.27)
SDQ—Conduct problems	1.25 (1.11)	1.75 (1.45)	1.62 (1.52)	2.04 (1.49)	2 (1.49)	1.49 (1.55)
SDQ—Hyperactivity/inattention	3.12 (2.15)	2.64 (1.87)	2.99 (2.1)	3.78 (2.37)	4.28 (2.5)	3.13 (2.18)
SDQ—Peer relationship problems	1.29 (1.5)	1.13 (1.34)	1.38 (1.55)	2.13 (1.68)	1.69 (1.62)	2.21 (1.68)
SDQ—Prosocial	8.54 (1.53)	8.43 (1.67)	8.09 (1.68)	7.63 (1.8)	8.51 (1.62)	8.34 (1.7)
**Student**						
SSIS SEL*b* SEL composite	44.3 (6.61)	41.89 (7.53)	39.22 (7.82)	41.23 (7.14)	44.55 (7.76)	47.19 (6.91)
SDQ—Emotional symptoms	3.49 (2.47)	3.49 (2.59)	3.49 (2.67)	3.63 (2.46)	4.13 (2.24)	3.79 (2.56)
SDQ—Conduct problems	1.7 (1.31)	2.37 (1.54)	2.08 (1.76)	2.12 (1.47)	2.15 (1.71)	1.77 (1.57)
SDQ—Hyperactivity/inattention	3.59 (2.19)	3.47 (2.42)	3.84 (2.29)	3.86 (2.13)	4.37 (2.32)	3.32 (2.15)
SDQ—Peer relationship problems	2.05 (1.73)	1.94 (1.76)	2.01 (1.86)	2.83 (1.85)	2.08 (1.68)	2.52 (1.65)
SDQ—Prosocial	8.12 (1.76)	8.02 (1.65)	7.61 (1.8)	7.27 (1.76)	8.11 (1.79)	8.33 (1.54)
CD—Resilience	28.02 (5.48)	25.88 (6.85)	21.97 (7.95)	25.08 (6.56)	25.03 (7.2)	26.92 (6.99)

SDQ, Strengths and Difficulties Questionnaire; CD, Connor Davidson Resilience Scale. SSIS SELb scores range from 0 to 60. SDQ subscale scores range from 0 to 10. CD scores range from 0 to 40.

**TABLE 5:** Means and standard deviations for predictive validity samples across country.

Scale	Croatia		Greece		Italy		Latvia		Portugal		Romania	
						
	Pre	Post	Pre	Post	Pre	Post	Pre	Post	Pre	Post	Pre	Post
**Teacher**												
SSIS SEL*b* SEL composite	41.65 (10.38)	43.03 (9.98)	43.11 (9.87)	43.06 (10.79)	38.36 (11.62)	39.06 (11.6)	41.46 (10.36)	42.94 (10.31)	44.14 (9.60)	43.88 (9.83)	42.91 (11.83)	44.65 (12.07)
SDQ—Emotional symptoms	1.39 (1.91)	1.43 (1.9)	1.54 (1.95)	1.41 (1.87)	1.83 (1.98)	1.64 (1.92)	1.81 (2.00)	1.67 (1.87)	2.44 (2.25)	2.28 (2.18)	1.79 (1.96)	1.90 (2.15)
SDQ—Conduct problems	0.90 (1.33)	1.06 (1.61)	0.92 (1.39)	1.00 (1.45)	1.21 (1.77)	1.32 (1.79)	1.39 (1.77)	1.34 (1.76)	1.29 (1.56)	1.42 (1.64)	1.04 (1.56)	1.05 (1.62)
SDQ—Hyperactivity/inattention	2.93 (2.72)	2.87 (2.74)	3.01 (2.73)	2.92 (2.68)	2.92 (2.65)	2.81 (2.66)	3.24 (2.82)	3.10 (2.70)	3.17 (2.70)	3.30 (2.70)	2.55 (2.58)	2.31 (2.51)
SDQ—Peer relationship problems	1.62 (1.92)	1.45 (1.83)	1.60 (1.73)	1.64 (1.69)	1.62 (1.8)	1.49 (1.66)	2.17 (1.99)	2.12 (1.89)	1.50 (1.76)	1.77 (1.78)	2.05 (1.66)	1.76 (1.59)
SDQ—Prosocial	7.74 (2.28)	7.97 (2.28)	7.28 (2.3)	7.11 (2.52)	6.95 (2.48)	7.03 (2.39)	6.97 (2.25)	6.98 (2.34)	7.56 (2.23)	7.41 (2.38)	7.84 (2.20)	8.08 (2.12)
**Parent**												
SSIS SEL*b* SEL composite	42.25 (7.63)	43.71 (7.93)	45.02 (7.21)	45.24 (9.31)	42.52 (7.35)	43.25 (7.80)	37.09 (9.03)	38.20 (9.11)	44.56 (7.44)	45.03 (8.01)	44.48 (8.16)	45.18 (8.33)
SDQ—Emotional symptoms	1.18 (1.46)	1.31 (1.65)	1.83 (1.76)	1.73 (1.77)	2.19 (1.95)	2.14 (2.02)	2.23 (1.92)	2.13 (2.00)	2.88 (2.14)	2.68 (2.08)	2.68 (2.11)	2.38 (2.11)
SDQ—Conduct problems	1.31 (1.12)	1.31 (1.33)	1.65 (1.39)	1.60 (1.34)	1.58 (1.48)	1.55 (1.43)	2.02 (1.48)	2.00 (1.49)	1.96 (1.47)	1.94 (1.54)	1.40 (1.42)	1.33 (1.44)
SDQ—Hyperactivity/inattention	3.18 (2.2)	3.04 (2.24)	2.66 (1.93)	2.81 (2.24)	2.91 (2.03)	2.80 (1.98)	3.77 (2.42)	3.73 (2.41)	4.39 (2.53)	4.09 (2.56)	2.97 (2.07)	2.76 (2.04)
SDQ—Peer relationship problems	1.37 (1.54)	1.37 (1.61)	1.31 (1.51)	1.36 (1.51)	1.28 (1.61)	1.38 (1.66)	2.15 (1.68)	2.11 (1.70)	1.68 (1.66)	1.58 (1.61)	2.13 (1.65)	2.01 (1.66)
SDQ—Prosocial	8.53 (1.55)	8.52 (1.52)	8.12 (2.06)	8.11 (2.26)	8.07 (1.75)	8.09 (1.84)	7.68 (1.80)	7.56 (1.82)	8.51 (1.63)	8.64 (1.61)	8.28 (1.65)	8.42 (1.62)
**Student**												
SSIS SEL*b* SEL composite	44.12 (6.08)	45.22 (6.52)	38.89 (7.77)	41.38 (6.69)	38.62 (6.23)	39.12 (6.66)	40.67 (7.41)	39.93 (7.83)	44.02 (7.98)	44.15 (7.61)	45.84 (7.38)	46.67 (7.47)
SDQ—Emotional symptoms	3.51 (2.28)	3.71 (2.4)	3.70 (2.93)	2.70 (2.27)	3.68 (2.70)	3.86 (2.56)	3.96 (2.34)	3.85 (2.54)	4.15 (2.30)	4.37 (2.37)	3.80 (2.45)	3.45 (2.75)
SDQ—Conduct problems	1.67 (1.23)	1.57 (1.17)	2.94 (1.66)	2.34 (1.59)	1.98 (1.53)	1.80 (1.53)	2.15 (1.50)	2.27 (1.58)	1.95 (1.63)	1.91 (1.73)	2.00 (1.37)	1.71 (1.57)
SDQ–Hyperactivity/inattention	3.49 (2.19)	3.78 (2.08)	4.04 (2.59)	3.04 (2.07)	4.07 (2.14)	3.62 (2.04)	3.94 (1.95)	4.44 (2.19)	4.45 (2.40)	4.48 (2.47)	3.64 (2.38)	3.18 (2.03)
SDQ—Peer relationship problems	2.22 (1.87)	2.00 (1.6)	2.74 (2.33)	1.87 (1.36)	2.26 (1.89)	2.07 (1.89)	3.05 (1.94)	3.09 (1.87)	1.90 (1.51)	2.10 (1.73)	2.44 (1.70)	2.40 (1.74)
SDQ—Prosocial	7.82 (1.88)	8.00 (1.77)	7.60 (1.99)	8.00 (1.35)	7.39 (1.64)	7.60 (1.71)	7.25 (1.75)	6.97 (1.95)	8.07 (1.80)	8.03 (1.75)	8.33 (1.69)	8.33 (1.71)
CD—Resilience	28.31 (6.16)	27.67 (5.67)	24.79 (7.14)	25.66 (6.27)	21.84 (7.40)	22.04 (8.22)	24.56 (6.69)	25.01 (6.93)	25.44 (7.29)	24.81 (7.59)	27.25 (8.01)	27.69 (7.14)

**TABLE 6:** Validity coefficients for SSIS SEL*b* composite across country.

Scale	Croatia		Greece		Italy		Latvia		Portugal		Romania	
						
	C	P	C	P	C	P	C	P	C	P	C	P
**Teacher**												
SDQ—Emotional symptoms	−0.32	−0.18	−0.43	−0.31	−0.38	−0.26	−0.36	−0.20	−0.23	−0.24	−0.46	−0.54
SDQ—Conduct problems	−0.61	−0.47	−0.58	−0.51	−0.65	−0.54	−0.65	−0.53	−0.59	−0.45	−0.61	−0.57
SDQ—Hyperactivity/inattention	−0.66	−0.58	−0.67	−0.67	−0.71	−0.63	−0.69	−0.60	−0.64	−0.53	−0.70	−0.69
SDQ—Peer relationship problems	−0.47	−0.37	−0.49	−0.42	−0.42	−0.32	−0.53	−0.44	−0.44	−0.41	−0.54	−0.51
SDQ—Prosocial	0.77	0.59	0.76	0.61	0.76	0.60	0.75	0.60	0.65	0.55	0.75	0.61
**Parent**												
SDQ—Emotional symptoms	−0.23	−0.19	−0.24	−0.11^ns^	−0.24	−0.10^†^	−0.33	−0.24	−0.19	−0.16	−0.32	−0.23
SDQ—Conduct problems	−0.52	−0.31	−0.48	−0.27	−0.49	−0.36	−0.52	−0.41	−0.50	−0.37	−0.52	−0.32
SDQ—Hyperactivity/inattention	−0.45	−0.39	−0.44	−0.24	−0.47	−0.30	−0.48	−0.40	−0.45	−0.31	−0.52	−0.39
SDQ—Peer relationship problems	−0.32	−0.16	−0.28	−0.33	−0.28	−0.28	−0.35	−0.29	−0.29	−0.25	−0.28	−0.06^ns^
SDQ—Prosocial	0.65	0.50	0.58	0.40	0.56	0.51	0.62	0.65	0.44	0.36	0.59	0.35
**Student**												
SDQ—Emotional symptoms	−0.07^ns^	−0.15^ns^	−0.24	−0.43	−0.13	0.10^ns^	−0.18	0.02^ns^	−0.05^ns^	−0.15	−0.28	−0.19^ns^
SDQ—Conduct problems	−0.47	−0.24^†^	−0.47	−0.05^ns^	−0.41	−0.16^†^	−0.42	−0.26	−0.40	−0.38	−0.49	−0.30^†^
SDQ—Hyperactivity/inattention	−0.19^ns^	−0.03^ns^	−0.43	−0.26^†^	−0.43	−0.10^ns^	−0.44	−0.24	−0.45	−0.39	−0.49	−0.30
SDQ—Peer relationship problems	−0.40	−0.28	−0.32	−0.19^ns^	−0.26	0.02^ns^	−0.29	−0.17	−0.37	−0.29	−0.34	−0.27
SDQ—Prosocial	0.65	0.47	0.50	0.30	0.60	0.42	0.62	0.45	0.54	0.31	0.61	0.42
CD—Resilience	0.41	0.49	0.47	0.19^ns^	0.47	0.11^ns^	0.42	0.24	0.44	0.31	0.55	0.47

SDQ, Strengths and Difficulties Questionnaire; CD, Connor Davidson Resilience Scale; C, concurrent; P, predictive. Unless otherwise noted, all coefficients were statistically significant (p < 0.05). ns, not significant and †p < 0.10.

**TABLE 7:** Statistically significant validity correlation differences across country.

	Croatia	Greece	Italy	Latvia	Portugal	Romania
**SSIS SEL*b* teacher**						
Croatia	–					SDQ-ES <
Greece	SDQ-ES >	–				SDQ-ES <
Italy			–			SDQ-ES <
Latvia			SDQ-PRP >	–		
Portugal	SDQ-ES < SDQ-PRO <	SDQ-ES < SDQ-PRO <	SDQ-ES < SDQ-PRO <	SDQ-ES < SDQ-PRO <	–	SDQ-ES <
Romania	SDQ-ES >		SDQ-ES > SDQ-PRP >	SDQ-ES >	SDQ-ES > SDQ-PRP >	–
**SSIS SEL*b* parent**						
Croatia	–					
Greece		–		SDQ-PRO <		
Italy			–			
Latvia				–	SDQ-PRO >	SDQ-PRP > SDQ-PRO >
Portugal	SDQ-PRO <		SDQ-PRO <	SDQ-ES < SDQ-PRO <	–	
Romania					SDQ-ES > SDQ-PRO >	–
**SSIS SEL*b* student**						
Croatia	–					
Greece		–	SDQ-ES >	SDQ-ES >		
Italy			–		SDQ-PRP <	
Latvia				–		
Portugal		SDQ-ES <			–	
Romania			SDQ-ES >		SDQ-ES >	–

Concurrent correlations below the diagonal; predictive correlations above the diagonal.

≥ = Country in row greater has higher magnitude correlation (regardless of sign).

≤ = Country in column has higher magnitude correlation (regardless of sign).

SDQ, Strengths and Difficulties Questionnaire; ES, Emotional Symptoms subscale; PRO, Prosocial Behavior subscale; PRP, Peer Relationship Problems subscale.

### SSIS SEL*b* teacher

First, we completed correlational analyses for concurrent validity correlations. Regarding SSIS SEL*b* Composite—SDQ correlations, correlations generally were negative and medium for the SDQ Emotional Symptoms scale (−0.46 < *r* < −0.23; *Mdn.* = −0.37); negative and large for the SDQ Conduct Problems scale (−0.65 < *r* < −0.58; *Mdn.* = −0.61) and the Hyperactivity/Inattention scale (−0.71 < *r* < −0.64; *Mdn.* = −0.68); negative and generally medium/large for the SDQ Peer Relationship Problems scale (−0.54 < *r* < −0.42; *Mdn.* = −0.48); and positive and strong for the SDQ Prosocial scale (0.65 < *r* < 0.77; *Mdn.* = 0.76). All correlations were significant (*p* < 0.05). Correlations were statistically significantly different across countries for the SDQ Emotional Symptoms, SDQ Peer Relationship Problems, and SDQ Prosocial scales, but were not statistically significantly different for the SDQ Conduct Problems and SDQ Hyperactivity/Inattention scales. For the SDQ Emotional Symptoms scale, the correlation for Romania (*r* = −0.46) was stronger in magnitude relative to Croatia (*r* = −0.32), Italy (*r* = −0.38), Latvia (*r* = −0.35), and Portugal (*r* = −0.23). The correlation for Portugal also was weaker than Croatia, Greece (*r* = −0.43), Italy, and Latvia. Finally, the correlation for Greece was stronger in magnitude relative to Croatia. No other correlations were statistically different across countries.

Regarding predictive validity correlations, patterns mirrored concurrent validity correlations, but as expected were weaker in magnitude. Specifically, correlations between the SSIS SEL*b* SEL Composite and SDQ scales were negative and small to medium for the SDQ Emotional Symptoms scale (−0.54 < *r* < −0.18; *Mdn.* = −0.25); negative and generally large for the SDQ Conduct Problems (−0.57 < *r* < −0.45; *Mdn.* = −0.52) and Hyperactivity/Inattention (−0.69 < *r* < −0.53; *Mdn.* = −0.62) scales; negative and generally medium for the SDQ Peer Relationship Problems scale (−0.51 < *r* < −0.32; *Mdn.* = −0.42); and positive and strong for the SDQ Prosocial scale (0.55 < *r* < 0.61; *Mdn.* = 0.60). As with concurrent validity correlations, all correlations were significant (*p* < 0.05). Predictive correlations were not statistically significantly different for SDQ Conduct Problems, SDQ Hyperactivity/Inattention, and SDQ Peer Relationship Problems, and SDQ Prosocial scales, but correlations were statistically different across countries for the SDQ Emotional Symptoms scale. For this scale, the correlation between the SSIS SEL*b* SEL Composite and SDQ Emotional Symptoms scores was statistically significantly stronger for Romania (*r* = −0.54) compared with all countries including Croatia (*r* = −0.18), Greece (*r* = −0.31), Italy (*r* = −0.26), Latvia (*r* = −0.20), and Portugal (*r* = −0.24). No other correlations were statistically significantly different across countries.

### SSIS SEL*b* parent

We replicated correlational analyses for the SSIS SEL*b* Parent. For concurrent validity correlations, SSIS SEL*b* SEL Composite and SDQ scores were negative and generally small for SDQ Emotional Symptoms scores (−0.33 < *r* < −0.19; *Mdn.* = −0.24); negative and generally strong for SDQ Conduct Problems scores (−0.52 < *r* < −0.48; *Mdn.* = −0.51); negative and generally medium for SDQ Hyperactivity/Inattention scores (−0.52 < *r* < −0.44; *Mdn.* = −0.46); negative and generally small for SDQ Peer Relationship Problems scores (−0.35 < *r* < −0.28; *Mdn.* = −0.29); and positive and generally strong for SDQ Prosocial scores (0.44 < *r* < 0.65; *Mdn.* = 0.59). All correlations were significant (*p* < 0.05). Correlations were not statistically different across countries for SDQ Conduct Problems; SDQ Hyperactivity/Inattention; and SDQ Peer Relationship Problems scales. For SDQ Emotional Symptoms, the correlation for Portugal (*r* = −0.19) was statistically significantly weaker than both Latvia (*r* = −0.33) and Romania (*r* = −0.32), but no other correlations were statistically different across countries. For the SDQ Prosocial scale, SEL Composite correlations were weaker for Portugal (*r* = 0.44) relative to Croatia (*r* = 0.65), Italy (*r* = 0.56), Latvia (*r* = 0.62), and Romania (*r* = 0.59) but no other correlations were statistically significantly different across countries.

Regarding predictive validity correlations, SSIS SEL*b* SEL Composite—SDQ correlations were negative and small for SDQ Emotional Symptoms scores (−0.24 < *r* < −0.16; *Mdn.* = −0.21); negative and generally medium for SDQ Conduct Problems (−0.41 < *r* < −0.27; *Mdn.* = −0.34) and SDQ Hyperactivity/Inattention scores (−0.40 < *r* < −0.24; *Mdn.* = −0.35); negative and generally small for SDQ Peer Relationship Problems scores (−0.33 < *r* < −0.06; *Mdn.* = −0.27); and positive and generally medium for SDQ Prosocial scores (0.35 < *r* < 0.65; *Mdn.* = 0.45). A few of these correlations were either marginally statistically significant (*p* < 0.10) or not statistically significant (see Table 6). Regarding cross-country correlation differences, correlations were not statistically significantly different for correlations involving the SDQ Emotional Problems, SDQ Conduct Problems, and SDQ Hyperactivity/Inattention scales. Although the omnibus test for correlation differences involving the SDQ Peer Relationship Problems scale was statistically significant (*p* = 0.013), after application of the Benjamini-Hochberg procedure, only one individual country comparison was statistically significant. Specifically, the correlation for Romania (*r* = 0.06) was statistically weaker than the correlation for Latvia (*r* = −0.29). Correlations also differed across countries for the SDQ Prosocial scale. Specifically, the correlation for Latvia (*r* = 0.65) was statistically larger than Greece (*r* = 0.40), Portugal (*r* = 0.36) and Romania (*r* = 0.35), but no other correlations were different across countries.

### SSIS SEL*b* student

Finally, we completed analyses for the SSIS SEL*b* SEL Student Form Composite. Regarding concurrent validity correlations with outcome measures, we found correlations were negative and generally small for the SDQ Emotional Symptoms subscale (−0.28 < *r* < −0.05; *Mdn.* = −0.18), negative and medium for the SDQ Conduct Problems subscale (−0.49 < *r* < −0.40; *Mdn.* = −0.45); negative and generally medium for the SDQ Hyperactivity/Inattention subscale (−0.49 < *r* < −0.43; *Mdn.* = −0.44) and the SDQ Peer Relationship Problems scale (−0.40 < *r* < −0.26; *Mdn.* = −0.33); positive and strong for the SDQ Prosocial sub scale (0.50 < *r* < 0.65; *Mdn.* = 0.61); and positive and generally medium for the CD-RISC Composite (0.41 < *r* < 0.55; *Mdn.* = 0.46). Three of these correlations were not statistically significant, but the remaining were (*p* < 0.05). Across countries, most correlations were not statistically different (SDQ Conduct Problems; SDQ Hyperactivity/Inattention; SDQ Peer Relationship Problems; SDQ Prosocial; CD-RISC Composite). Correlations were statistically different across countries, however, for the SDQ Emotional Symptoms correlations. Specifically, the correlation for Portugal (*r* = −0.05) was statistically smaller relative to Greece (*r* = −0.24) and Romania (*r* = −0.28). Further, the correlation for Romania, was also stronger in magnitude relative to Italy (*r* = −0.12), but no other correlations were statistically significantly different across countries. All correlations are presented in Table 6.

Regarding predictive validity correlations, we found the correlations were negative and generally small for the SDQ Emotional Symptoms scale (−0.43 < *r* < 0.10; *Mdn.* = −0.15); negative and generally small for the SDQ Conduct Problems scale (−0.38 < *r* < −0.05; *Mdn.* = −0.25), the SDQ Hyperactivity/Inattention scale (−0.39 < *r* < −0.03; *Mdn.* = −0.25), and the SDQ Peer Relationship Problems scale (−0.29 < *r* < 0.02; *Mdn.* = −0.23); positive and medium for the SDQ Prosocial scale (0.30 < *r* < 0.47; *Mdn.* = 0.42); and positive and small to medium for the CD-RISC Composite (0.11 < *r* < 0.49; *Mdn.* = 0.28). Many of these correlations were marginally statistically significant (*p* < 0.10) or not statistically significant (see Table 6), especially for Greece and Italy. Regarding cross-country differences, correlations were not statistically significantly different across countries for the SDQ Conduct Problems, SDQ Hyperactivity/Inattention, SDQ Prosocial, and CD-RISC Composite scores. Correlations did differ across countries for the SDQ Emotional Symptoms and SDQ Peer Relationship Problems scales. For the SDQ Emotional Symptoms scale, the correlation for Greece (*r* = −0.43) was statistically larger in magnitude than the correlation for Italy (*r* = 0.10) and Latvia (*r* = 0.02), but no other correlations were statistically significantly different across country. For the SDQ Peer Relationship Problems scale, the correlation for Portugal (*r* = −0.29) was statistically larger in magnitude than the correlation for Italy (*r* = 0.02), but no other correlations were statistically significantly different across countries.

## Discussion

This study was undertaken to examine concurrent and predictive validity evidence for the Composite scores from the translated versions of the multi-informant SSIS SEL*b* Scales. This universal screening scale was developed in the United States and based on the CASEL five competency framework (CASEL, 2015). Specifically, the teacher, student, and parent forms of this assessment of children’s social emotional learning were translated as part of an investigation of the effectiveness of a mental health program (PROMEHS) delivered in schools across six European countries.

Using existing translated versions of the SDQ, a widely used multi-informant rating scale of children’s prosocial and problem behavior, and the 10-item student CD-RISC, a self-report measure of resilience behaviors, assessment results for large and representative samples of children were used to provide insights regarding the theoretical construct of SEL competency and validity of scores from translated versions of the SSIS SEL*b* Scales. This construct and social behavior representative of it was the central outcome variable of the PROMEHS project and expected to be associated with children’s mental health and school success.

### Major findings

As expected based on our guiding theory about the development and relevance of SEL competences to children’s mental health and schooling, we found substantial evidence, both concurrent and predictive, to support that SDQ Prosocial scores of students, as rated by parents, teachers, and students, were correlated positively and moderately with the SSIS SEL*b* SEL Composite. Specifically, the validity coefficients were highly consistent across the six participating countries and three informant types, with (a) concurrent correlations always stronger than their corresponding predictive correlations and (b) nearly all these correlations in the moderate to high range. These findings provide strong evidence of the convergent validity of scores from the translated SSIS SEL*b* scales.

Second, when SDQ difficulties subscales scores completed by the same raters were correlated with SEL*b* Composite scores, the evidence supported the supposition of moderate negative relations for both the concurrent and predictive results. Again, the expected pattern was found between concurrent and predictive correlations across informants from each country. Specifically, these validity coefficients were always negative, indicating the nature of the constructs being measured are different. In fact, each of the four SDQ problem scales—Emotional Symptoms, Conduct Problems, Hyperactivity/Inattention, and Peer Relationship Problems—when completed by each type of informant consistently correlated negatively with the corresponding SEL composite score. The magnitude of these negatively related constructs was generally in the low to moderate range, however, with the teacher informants these same validity coefficients generally ranged higher. These levels of magnitude between pairs of informants are typical (e.g., Elliott et al., 2020).

Finally, there was substantial evidence from students’ self-ratings to support the supposition that scores from the 10-item CD-RISC correlated moderately positively with the Composite SEL scores on the SEL*b*. Again, across all countries (a) the concurrent validity coefficients were higher in magnitude than the predictive coefficients and (b) nearly all the indicators of the relations between the SEL and resilience scores were in the moderate range.

### Additional findings

Taken as a whole, these findings supported our expectations in most cases. The direction and magnitude of these validity correlations provides much needed convergent and discriminant validity evidence for composite scores from a brief SEL measure translated for use across multiple European countries. With the large number of correlations computed, there were some individual correlations that fell outside the expected magnitude range. For example, the correlation between the SSIS SEL*b* SEL Composite and the SDQ Emotional Symptoms scale for the Croatian student sample was 0.07, which would be considered negligible (Cohen, 1988). Most of these correlations occurred for student respondents, and the trend was especially present for predictive validity correlations. Considering longstanding indications that, due to their still-developing introspective and self-awareness skills, students tend to be less reliable reporters than their parents and teachers (e.g., Jenkins et al., 2014; Anthony et al., 2020a,b), this pattern is not surprising. It does suggest that students’ self-ratings should be interpreted with these considerations in mind.

Another unanticipated observed pattern was that, in general, correlations were stronger in magnitude for SDQ Conduct Problems and Hyperactivity/Inattention scales relative to SDQ Emotional Symptoms and Peer Relationship Problems scales. This pattern held across informants and countries but was especially strong for parents and teachers. This finding is partially explainable by the tendency of external raters to be better able to rate more observable externalizing behaviors than internalizing behaviors or more subtle social behaviors to which they have little access (e.g., Dowdy et al., 2013). Yet, the fact that it held for student raters as well could also point to differential relations with negative behaviors and outcomes that could be important foundations of future validation work for the SSIS SEL*b* and similar measures. Future research is warranted.

Our cross-country comparisons also yielded some interesting and potentially important results. The most striking trend was that validity evidence, while strong in an absolute sense, tended to be weaker in magnitude for the Portuguese SSIS SEL*b* and stronger in magnitude for the Romanian SSIS SEL*b*. These patterns held only for teacher and parent informants and did not seem to be present for students. Although these patterns could indicate problems with the translation of the Portuguese SSIS SEL*b*, this seems unlikely because the differential validity relationships were only present for the SDQ Emotional Symptoms, Prosocial, and, to a lesser extent, Peer Relationship Problems subscales. It is possible that these SDQ scales have some translation or validity issues that need to be resolved with further research. Furthermore, it is possible that cultural differences in Portugal and Romania explain the weaker relationship between SEL and these social constructs in Portugal and the stronger relationship between them in Romania. Future cross-cultural research is warranted to better understand the nature and importance of SEL across European cultures.

Finally, although it was not the goal of this study to consider the reliability of these translated scales, it bears mentioning that in generally, the reliability of scores from the SSIS SEL*b* were strong and would support widespread research applications and, with further research and development, applied practice as well. A notable exception to this conclusion was for the Greek SSIS SEL*b*-S, which showed notably lower test-retest reliability than SSIS SEL*b* scores from other informants or countries. This finding held for other informants in Greece (parents) and for other scales with Greek students (both the SDQ and the CD-RISC), however, so it may have been an idiosyncratic feature of data collection of this study. Regardless, further research is necessary to support the use of the SSIS SEL*b* in Greece.

### Limitations and future research

Despite the promising psychometric evidence for the translated SSIS-SEL*b*, the results should be interpreted with caution due to some inherent limitations. First, it is important to note that these data were gathered during the COVID-19 pandemic and the social disruptions and other effects of that pandemic may have affected results. Next, reliability evidence for some of the translated external criterion measures was relatively weak both in the current sample and in prior literature. As weaknesses in the criterion measures could undermine the accuracy of some conclusions regarding validity coefficients from the current study, future studies should employ additional external criterion measures to further validate scores from the translated SSIS-SEL*b*. In this vein, it is important to note that all validity correlations reported in this study suffer from common method bias because validity analyses were conducted within informant. Considering that most cross-informant correlations are modest at best, future work should utilize external criterion measures such as discipline data, mental health service utilization data, and other similar sources of information to further validate scores from the SSIS SEL*b*. Another possible avenue for further validation work involves extant group studies. For example, it is well-established that students’ gender is associated with their SEL skills (e.g., Romer et al., 2011) with girls tending to be rated as having higher levels of SEL skills relative to boys. Extant group studies could evaluate whether this pattern and other known patterns across extant groups (e.g., disability status; socioeconomic status; etc.) holds with scores from translations of the SSIS SEL*b* to further support the validity of scores from these measures.

Relatedly, although the validity data from the study provide promising evidence for score inferences, more data are needed to support specific applications in European schools. Because the SSIS SEL*b* was created primarily to be a universal screener, conditional probability analyses might be a most profitable next step, but other data such as acceptability data, change-responsiveness data, and base rate data would be beneficial as well. Similarly, although the SSIS SEL*b* is not scored based on norm-referenced score interpretation, further work evaluating whether its criterion-referenced scoring approach is cross-culturally equivalent would greatly support the use of the SSIS SEL*b* in the included European countries.

## Conclusion

The results from this multi-country, multi-informant study with translated versions of the SSIS SEL*b* Scales provide support for the validity of their score inferences. In fact, the patterns of convergent and predictive validity indices for these translated measures of social emotional learning were consistent with our theoretical model of SEL competence, conformed to the research expectations, and were quite consistent across the six European countries with a diverse sample of children. Although additional research is necessary regarding specific applications of each translated version of the SSIS SEL*b* Scales, concurrent and predictive relations provide promising evidence for the validity of the Composite scores across multiple informants.

## Data availability statement

The raw data supporting the conclusions of this article will be made available by the authors, without undue reservation.

## Ethics statement

The studies involving human participants were reviewed and approved by the University of Milano-Bicocca, Italy; University of Rijeka, Croatia, University of Latvia, Latvia; University of Lisbon, Portugal; University of Patras, Greece; Stefan cel Mare University, Suceava. Written informed consent to participate in this study was provided by the participants’ legal guardian/next of kin.

## Author contributions

CA conceptualized the manuscript, conducted all statistical analyses, and drafted several sections of the manuscript as well as the tables. SE facilitated conceptualization, drafted the sections “Introduction” and “Discussion,” and revised the entire manuscript. MY facilitated conceptualization, drafted a large section of the “Introduction” and a table, and revised the manuscript. P-WL consulted on statistical analyses and revised the manuscript. JD revised the manuscript and wrote the conclusion of the manuscript. CC, LC, and PB selected the instruments, devised the research design, arranged and cleaned the data, and participated in the design and revision of the manuscript. IG, VO, VC, and EC contributed to the overall coordination of the project, to data collection, and to the revision of the manuscript. SV, MP, BM, CS, and AC contributed to the data collection and to the revision of the manuscript. All authors contributed to the article and approved the submitted version.

## Footnotes

1
https://www.promehs.org

2 To clarify, students whom teachers considered to be disadvantaged (or those from any other minority group) were neither specifically targeted nor excluded from the study, but were included based on application of our systematic sampling procedure.

## References

[B1] Anjos J. F. Dos Santos M. J. H. Ribeiro M. T. Moreira S. (2019). Connor-davidson resilience scale: validation study in a portuguese sample. *BMJ Open* 9:e026836. 10.1136/bmjopen-2018-026836 31253616 PMC6609049

[B2] Anthony C. J. Brann K. Elliott S. N. Garis E. J. (2022). Examining the structural validity of the SSIS SEL brief scales–teacher and student forms. *Psychol. Sch.* 59 260–280. 10.1002/pits.22607

[B3] Anthony C. J. Elliott S. N. DiPerna J. C. Lei P. W. (2020a). The SSIS SEL brief scales–student form: initial development and validation. *Sch. Psychol.* 35:277. 10.1037/spq0000390 32673055

[B4] Anthony C. J. Elliott S. N. DiPerna J. C. Lei P. (2020b). Multirater assessment of young children’s social and emotional learning *via* the SSIS SEL brief scales–preschool forms. *Early Child. Res. Q.* 53 625–627. 10.1016/j.ecresq.2020.07.006

[B5] Anthony C. J. Elliott S. N. DiPerna J. C. Lei P. (2021). Initial development and validation of the SSIS SEL brief scales–teacher form. *J. Psychoeduc. Assess.* 39 166–181. 10.1177/0734282920953240

[B6] Benjamini Y. Hochberg Y. (1995). Controlling the false discovery rate: a practical and powerful approach to multiple testing. *J. R. Stat. Soc. Series B* 57 289–300. 10.1111/j.2517-6161.1995.tb02031

[B7] Bibou-Nakou I. Markos A. Padeliadu S. Chatzilampou P. Ververidou S. (2019). Multi-informant evaluation of students’ psychosocial status through SDQ in a national Greek sample. *Child. Youth Ser. Rev.* 96 47–54.

[B8] Campbell-Sills L. Stein M. B. (2007). Psychometric analysis and refinement of the ConnoreDavidson resilience scale (CD-RISC): validation of a 10-item measure of resilience. *J. Traum. Stress* 20 1019–1028. 10.1002/jts.20271 18157881

[B9] Caprara G. V. Barbaranelli C. Pastorelli C. Bandura A. Zimbardo P. G. (2000). Prosocial foundations of children’s academic achievement. *Psychol. Sci.* 11 302–306. 10.1111/1467-9280.00260 11273389

[B10] CASEL (2015). *Effective Social and Emotional Learning Programs: Middle and High School.* Chicago, IL: CASEL.

[B11] Cavioni V. Grazzani I. Ornaghi V. Pepe A. Pons F. (2020). Assessing the factor structure and measurement invariance of the Test of Emotion Comprehension (TEC): a large cross-sectional study with children aged 3-10 years. *J. Cogn. Dev.* 21 406–424. 10.1080/15248372.2020.1741365

[B12] Cefai C. Arlove A. Duca M. Galea N. Muscat M. Cavioni V. (2018a). RESCUR surfing the waves: an evaluation of a resilience programme in the early years. *Pastor. Care Educ*. 36, 189–204. 10.1080/02643944.2018.1479224

[B13] Cefai C. Bartolo P. A. Cavioni V. Downes P. (2018b). *Strengthening Social and Emotional Education as a Core Curricular Area Across the EU: A Review of the International Evidence. (NESET II report).* Luxembourg: Publications Office of the European Union.

[B14] Cefai C. Camilleri L. Bartolo P. Grazzani I. Cavioni V. Conte E. (in press). The effectiveness of a school-based, universal mental health programme in six European countries. *Front. Psychol.* 10.3389/fpsyg.2022.925614 PMC9393716 36003110

[B15] Cefai C. Simões C. Caravita S. (2021). *A Systemic, Whole-School Approach to Mental Health and Well-Being in Schools in the EU*. NESET Report, Luxembourg: Publications Office of the European Union.

[B16] Cohen J. (1988). *Statistical Power Analysis for the Behavioral Sciences*, 2nd Edn. Hillsdale, NJ: Earlbaum.

[B17] Collaborative for Academic, Social, and Emotional Learning [CASEL] (2021). *SEL: What are the Core Competence Areas and Where are They Promoted?* Available online at: https://casel.org/sel-framework/ (accessed April 22, 2022).

[B18] Connor K. M. Davidson J. R. (2003). Development of a new resilience scale: the connor-davidson resilience scale (CD-RISC). *Depress. Anxiety* 18 76–82. 10.1002/da.10113 12964174

[B19] Costa P. A. Tasker F. Ramos C. Leal I. (2020). Psychometric properties of the parent’s versions of the SDQ and the PANAS-X in a community sample of portuguese parents. *Clin. Child Psychol. Psychiatry* 25 520–532. 10.1177/1359104519891759 31793797

[B20] Di Riso D. Salcuni S. Chessa D. Raudino A. Lis A. Altoè G. (2010). The strengths and difficulties questionnaire (SDQ). Early evidence of its reliability and validity in a community sample of Italian children. *Pers. Indiv. Differ.* 49 570–575. 10.1016/j.paid.2010.05.005

[B21] DiPerna J. C. Lei P. Bellinger J. Cheng W. (2015). Efficacy of the social skills improvement system classwide intervention program (SSIS-CIP) primary version. *Sch. Psychol. Q.* 30:123. 10.1037/e615512013-001 25111465

[B22] DiPerna J. C. Lei P. Bellinger J. Cheng W. (2016). Effects of a universal positive classroom behavior program on student learning. *Psychol. Sch.* 53 189–203. 10.1002/pits.21891

[B23] DiPerna J. C. Lei P. Cheng W. Hart S. C. Bellinger J. (2018). A cluster randomized trial of the social skills improvement system-classwide intervention program (SSIS-CIP) in first grade. *J. Educ. Psychol.* 110 1–16. 10.1037/edu0000191

[B24] Dowdy E. Doane K. Eklund K. Dever B. (2013). A comparison of teacher nomination and screening to identify behavioral and emotional risk within a sample of underrepresented students. *J. Emot. Behav. Disord.* 21 127–137.

[B25] Durlak J. A. Weissberg R. P. Dymnicki A. B. Taylor R. D. Schellinger K. B. (2011). The impact of enhancing students’ social and emotional learning: a meta-analysis of school-based universal interventions. *Child Dev.* 82 405–432. 21291449 10.1111/j.1467-8624.2010.01564.x

[B26] Elliott S. N. Anthony C. J. DiPerna J. C. Lei P. W. Gresham F. M. (2020). *SSIS SEL Brief + Mental Health Scales - Student.* Scottsdale, AZ: SAIL Collaborative.

[B27] Elliott S. N. Anthony C. J. Lei P. W. DiPerna J. C. (2021). Efficient assessment of the whole social–emotional child: using parents to rate SEL competencies and concurrent emotional behavior concerns. *School Ment. Health* 13 392–405. 10.1007/s12310-021-09429-7

[B28] Elliott S. N. Lei P. W. Anthony C. J. DiPerna J. C. (in press). Screening the whole social-emotional child: integrating emotional behavior concerns to expand the utility of the SSIS SEL brief scales. *Sch. Psychol. Rev.* 10.1080/2372966X.2020.1857659

[B29] Giannakopoulos G. Dimitrakaki C. Papadopoulou K. Tzavara C. Kolaitis G. Ravens-Sieberer U. (2013). Reliability and validity of the strengths and difficulties questionnaire in Greek adolescents and their parents. *Health* 5: 39458.

[B30] Giurcă I. C. Baban A. Pintea S. Macavei B. (2021). Psychometric properties of the 25-item connor-davison resilience scale: preliminary data for a romanian military population. *Land Forces Acad. Rev.* 26 31–38. 10.2478/raft-2021-0005

[B31] Goodman R. (1997). The strengths and difficulties questionnaire: a research note. *J. Child Psychol. Psychiatry* 38 581–586. 9255702 10.1111/j.1469-7610.1997.tb01545.x

[B32] Grazzani I. Agliati A. Cavioni V. Conte E. Gandellini S. Lupica Spagnolo M. (2022). Adolescents’ resilience during COVID-19 pandemic and its mediating role in the association between SEL and mental health. *Front. Psychol.* 13:801761. 10.3389/fpsyg.2022.801761 35197901 PMC8860228

[B33] Gresham F. M. Elliott S. N. (2017). *Social Skills Improvement System Social Emotional Learning Edition Rating Forms.* Bloomington, MN: Pearson Assessments.

[B34] Hambleton R. K. Swaminathan H. Rogers H. J. (1991). *Fundamentals of Item Response Theory*, Vol. 2. Thousand Oaks, CA: Sage.

[B35] Jenkins L. N. Demaray M. K. Wren N. S. Secord S. M. Lyell K. M. Magers A. M. (2014). A critical review of five commonly used social-emotional and behavioral screeners for elementary or secondary schools. *Contemp. School Psychol.* 18 241–254. 10.1007/s40688-014-0026-6

[B36] Jones C. R. Barrett S. L. Bite I. Legzdina M. Arina K. Higgins A. (2020). Development of the signposting questionnaire for autism (SQ-A): measurement comparison with the 10-item autism spectrum quotient-child and the strengths and difficulties questionnaire in the UK and Latvia. *Mol. Autism* 11:64. 10.1186/s13229-020-00368-9 32799931 PMC7429457

[B37] Mahoney J. L. Weissberg R. P. Greenberg M. T. Dusenbury L. Jagers R. J. Niemi K. (2021). Systemic social and emotional learning: promoting educational success for all preschool to high school students. *Am. Psychol.* 76 1128–1142. 10.1037/amp0000701 33030926

[B38] Martinsone B. Supe I. Stokenberga I. Damberga I. Cefai C. Camilleri L. (2022). Social emotional competence, learning outcomes, emotional and behavioral difficulties of preschool children: parent and teacher evaluations. *Front. Psychol.* 12:760782. 10.3389/fpsyg.2021.760782 35185671 PMC8852736

[B39] Mikuš M. Škegro B. Karadjole V. S. Lešin J. Banović V. Herman M. (2020). Maternity blues among croatian mothers–a single-center study. *Psychiatr. Danub.* 33 342–346. 10.24869/psyd.2021.342 34795176

[B40] Muthén Muthén (1998-2019). *Mplus User’s Guide. Eighth Edition.* Los Angeles, CA: Muthén & Muthén.

[B41] Reyes J. A. Elias M. J. Parker S. J. Rosenblatt J. L. (2013). “Promoting educational equity in disadvantaged youth: the role of resilience and social-emotional learning,” in *Handbook of Resilience in Children*, eds Goldstein S. Brooks R. (Boston, MA: Springer).

[B42] Reynolds C. R. Kamphaus R. W. (2004). *Behavior Assessment for Children.* Circle Pines, MN: American Guidance Service.

[B43] Rodrigues S. Barbosa-Ducharne M. Del Valle J. F. Campos J. (2019). Psychological adjustment of adolescents in residential care: comparative analysis of youth self-report/strengths and difficulties questionnaire. *Child Adolesc. Soc. Work J.* 36 247–258. 10.1007/s10560-019-00614-x

[B44] Romer N. Ravitch N. K. Tom K. Merrell K. W. Wesley K. L. (2011). Gender differences in positive social–emotional functioning. *Psychol. Sch.* 48 958–970. 10.1002/pits.20604

[B45] Satorra A. Bentler P. M. (2001). A scaled difference chi-square test statistic for moment structure analysis. *Psychometrika* 66 507–514. 10.1007/BF02296192 PMC2905175 20640194

[B46] Sharratt K. Foca L. Gavriluta C. Jones A. Asiminei R. (2014). Dimensionality and construct validity of the Romanian self-report strengths and difficulties questionnaire (SDQ). *Romanian J. Appl. Psychol.* 16 33–39.

[B47] Stevanovic D. Urbán R. Atilola O. Vostanis P. Balhara Y. S. Avicenna M. (2015). Does the strengths and difficulties questionnaire–self report yield invariant measurements across different nations? Data from the international child mental health study group. *Epidemiol. Psychiatr. Sci.* 24 323–334. 10.1017/S2045796014000201 24785706 PMC7192188

[B48] Tatalović Vorkapič S. Slaviček M. Vlah N. (2017). Strengths and difficulties in Croatian preschool children: validation of the strengths and difficulties questionnaire. *Hrvatska Revija Za Rehabilitacijska Istraživanja* 53 231–243. 10.1037/t70468-000

[B49] Tobia V. Marzocchi G. M. (2018). The strengths and difficulties questionnaire-parents for Italian school-aged children: psychometric properties and norms. *Child Psychiatry Hum. Dev.* 49 1–8. 10.1007/s10578-017-0723-2 28391540

[B50] Tsigkaropoulou E. Douzenis A. Tsitas N. Ferentinos P. Liappas I. Michopoulos I. (2018). Greek version of the connor-davidson resilience scale: psychometric properties in a sample of 546 subjects. *In Vivo* 32 1629–1634. 10.21873/invivo.11424 30348726 PMC6365761

[B51] Veiga G. de Leng W. Cachucho R. Ketelaar L. Kok J. N. Knobbe A. (2017). Social competence at the playground: preschoolers during recess. *Infant and Child Dev.* 26:e1957. 10.1002/icd.1957

[B52] Yoder N. Posamentier J. Godek D. Seibel K. Dusenbury L. (2020). *State Efforts to Elevate Social and Emotional Learning During the Pandemic.* Available online at: https://casel.s3.us-east-2.amazonaws.com/state-efforts-elevate-social-emotional-learning-during-pandemic.pdf (accessed April 22, 2022).

