# Supplementary material for: Multi-informant validity evidence for the SSIS SEL Brief Scales across six European countries
Source: Front Psychol. 2022 Aug 2;13:928189. doi: 10.3389/fpsyg.2022.928189 (PMC9380595; doi:10.3389/fpsyg.2022.928189)
Supplement: Supplementary file 1 [file Data_Sheet_1.pdf]

Relevant Psychometric Research on Validity Measures

To best support the validity of score inferences from a translated measure, it is important that assessments used as criterion measures have well established validity evidence. The two criterion measures used in the current study, the Strengths and Difficulties Questionnaire (SDQ; Goodman, 1997) and the Connor-Davidson Resilience Scale (CD-RISC; Conner & Davidson, 2003), have extensive validity evidence across most combinations of participating informants and countries (Table 1).

Strengths and Difficulties Questionnaire

The SDQ (Goodman, 1997) is a behavioral screener used to assess the mental health of children and adolescents. This measure may be used to collect data from a child, parent, or teacher, using respective respondent forms of the SDQ. To support the use of the translated versions of the SDQ as a validity measure, research was examined to understand its psychometric properties as used in countries participating in the PROMEHS project. First, the Greek translations of all three respondent forms of the SDQ have been previously validated for use with Greek samples. For example, in a Greek study (Giannakopoulos et al., 2013), the SDQ was completed by a representative sample of 1194 adolescents and their parents. Results indicated that acceptable levels of internal consistency reliability were found, with a Cronbach α of 0.70 or higher for all subscales except for Peer Relationship Problems and Conduct Problems. These researchers also found significant correlations between the Greek translation of the SDQ and a related measure, KIDSCREEN, supporting the convergent validity of the SDQ scores.

Studies with the Italian translations of all three respondent forms have also supported the use of the SDQ with Italian populations. For example, the original five-factor model was confirmed for the parent and teacher forms of the Italian SDQ (Tobia, & Marzocchi, 2018). This

study reported Cronbach alpha values ranging from poor (α=0.56 for conduct problems) to good (α=0.81 for the total difficulties score). It was determined that Item 22 ("Steals from home, school or elsewhere") contributed most significantly to the lower reliability observed in the conduct problems subscale. In a separate study, the psychometric properties of the self-report Italian SDQ were found to support the use of some scales from this assessment with Italian children and adolescents (Di Riso et al., 2010).

Extensive research has also been conducted on the Latvian translations of the SDQ. Specifically, Martinsone et al. (2022) administered the Latvian translation of the SDQ to teachers and parents of preschool children in Latvia. When comparing results from respondent forms, it was revealed that parents tended to rate their children's prosocial behavior, conduct problems, hyperactivity, and total difficulties higher than the teacher report. However, both parents and teachers tended to rate emotional problems and peer problems similarly. The Latvian translations of the SDQ parent and teacher forms were also found to have satisfactory internal consistency.

Next, the psychometric properties of scores from the Portuguese SDQ also have been been extensively evaluated. For example, in a study conducted by Costa et al. (2020), a confirmatory factor analysis of the parent SDQ found evidence supporting the both a three-factor and five-factor structure structure of the SDQ. The researchers also found significant correlations between all subscales of the Portuguese SDQ. Further, discriminant validity and acceptable reliability were also confirmed for all factors scores assessed (Costa et al., 2020). Separately, the self-report SDQ was administered to a sample of Portuguese children living in residential care facilities, and results indicated that this measure correlated significantly with a similar assessment, the Youth Self-Report (YSR; Achenbach & Rescorla, 2001), indicating satisfactory convergent validity (Rodrigues et al., 2019). Results also indicated the total difficulties scale to

have a Cronbach alpha value of 0.64 and adequate subscale reliability (Rodrigues et al., 2019). Overall, the Portuguese translation of the SDQ has been found to have sufficient reliability and validity evidence for use with Portuguese samples.

Finally, although less research has been conducted in Romania relative to other countries participating in this study, Romanian translations of the parent, teacher, and self-report forms of the SDQ also have been examined. Lee et al. (2014) measured mental health outcomes among preadolescent Romanian children using the parent and teacher forms of the Romanian SDQ. Separately, Sharratt et al. (2014) examined the psychometric properties of the self-report Romanian SDQ and determined using a confirmatory factor analysis that a five-factor model fit the data best. Low internal consistency and low item loading, however, were observed for all subscales except prosocial behavior. First, in a study conducted in Croatia, a sample of 461 preschool children was assessed by 29 preschool teachers using the Croatian translation of the SDQ-Teacher Form. A confirmatory factor analysis indicated that five factors (Conduct Problems, Emotional Symptoms, Hyperactivity/Inattention, Prosocial Behaviors, and Peer Relationship Problems) were extracted, accounting for 57.35% of the total variance and supporting the factor structure of the original version of the SDQ. Further, moderate to high Cronbach alpha values (ranging from .63 to .84) were found across all subscales, supporting the use of the Croatian translation of the SDQ as a reliable measure for this population (Tatalović Vorkapić et al., 2017).

Connor-Davidson Resilience Scale

The CD-RISC (Conner & Davidson, 2003) has been translated for use in each of the six participating PROMEHS countries. Studies using these translations were reviewed; however, there is less research that specifically evaluates the psychometric properties of the translated

versions of the CD-RISC than the SDQ. Nevertheless, this measure has been used in various capacities across the six participating countries in the present study. For instance, Mikuš et al. (2020) used the Croatian translation of the CD-RISC in a cross-sectional study to assess outcomes related to maternity blues with a sample of 227 mothers. Findings from this study indicated that higher scores on the Stein's Maternity Blues Scale were significantly correlated with lower levels of resilience, supporting the validity of the Croatian CD-RISC scores. Further, research on the psychometric properties of the Greek version of the CD-RISC found high internal consistency (Cronbach's alpha=0.925) and high test-retest reliability (intra-class correlation coefficient=0.925) with samples of Greek university students and psychiatric patients (Tsigkaropoulou et al., 2019). The CD-RISC also has been translated into Italian and tested with various sample groups in Italy. Grazzani et al. (2022) used the CD-RISC to assess resilience in a sample of 778 Italian children and adolescents. Resilience had a significant positive correlation with SEL skills measured by the SSIS SEL*b*, supporting the convergent validity of the Italian CD-RISC scores (Grazzani et al., 2022).

In Portugal, the structural and convergent validity evidence of the Portuguese version of the CD-RISC was evaluated with 881 participants (Faria Anjos et al., 2019). Results from a confirmatory factor analysis found a three-factor structure, which does not reflect the five-factor structure presented by the creators of this measure but is consistent with more recent findings on the factor structure of the CD-RISC. This measure also demonstrated good convergent validity evidence, as subscales from the CD-RISC were significantly correlated with various related measures (Anjos et al., 2019). The construct validity evidence of the Romanian version of the CD-RISC was evaluated in a sample of Romanian military personnel (Giurcă et al., 2021). Findings revealed that the Romanian CD-RISC is a psychometrically sound measure with high

levels of internal consistency, supporting its use within the present study (Giurcă et al., 2021). Previous findings on the psychometric properties of the Latvian version of the CD-RISC were not found. However, a Latvian translation of the CD-RISC was created using forward-backward translation procedures.

Table S1.

Example Items from the SSIS SEL Brief Scales and Validity Measures

Scale	Teacher	Parent	Student
**SSIS SEL Brief Scales**			
Self-Awareness	Identifies own emotions accurately.	Evaluates social emotional strengths and weaknesses.	I ask for help when I need it.
Self-Management	Completes tasks without bothering others.	Stays calm when teased.	I pay attention when the teacher talks to the class.
Social Awareness	Shows concern for others.	Stands up for others who are treated unfairly.	I help my friends when they are having a problem.
Relationship Skills	Interacts well with other children.	Introduces themself to others.	I work well with my classmates.
Responsible Decision-Making	Takes responsibility for their own actions.	Respects the property of others.	I do the right thing without being told.
**Strengths and Difficulties Questionnaire**			
Emotional Symptoms	Often complains of headaches, stomach-aches or sickness	Many worries or often seems worried	I am often unhappy, down-hearted, or tearful
Conduct Problems	Often has temper tantrums or hot tempers	Often fights with other children or bullies them	I am often accused of lying or cheating
Hyperactivity/Inattention	Restless, overactive, cannot stay still for long	Constantly fidgeting or squirming	I am easily distracted, I find it difficult to concentrate
Peer Relationship Problems	Rather solitary, tends to play alone	Picked on or bullied by other children	I get on better with adults than with other people my own age
Prosocial	Considerate of other people’s feelings	Shares readily with other children for example toys, treats, pencils	I am kind to younger children
Connor-Davidson Resilience Scale	-	-	I am able to adapt when changes occur.

Figure S1.

Diagram of Analytic Model Tested

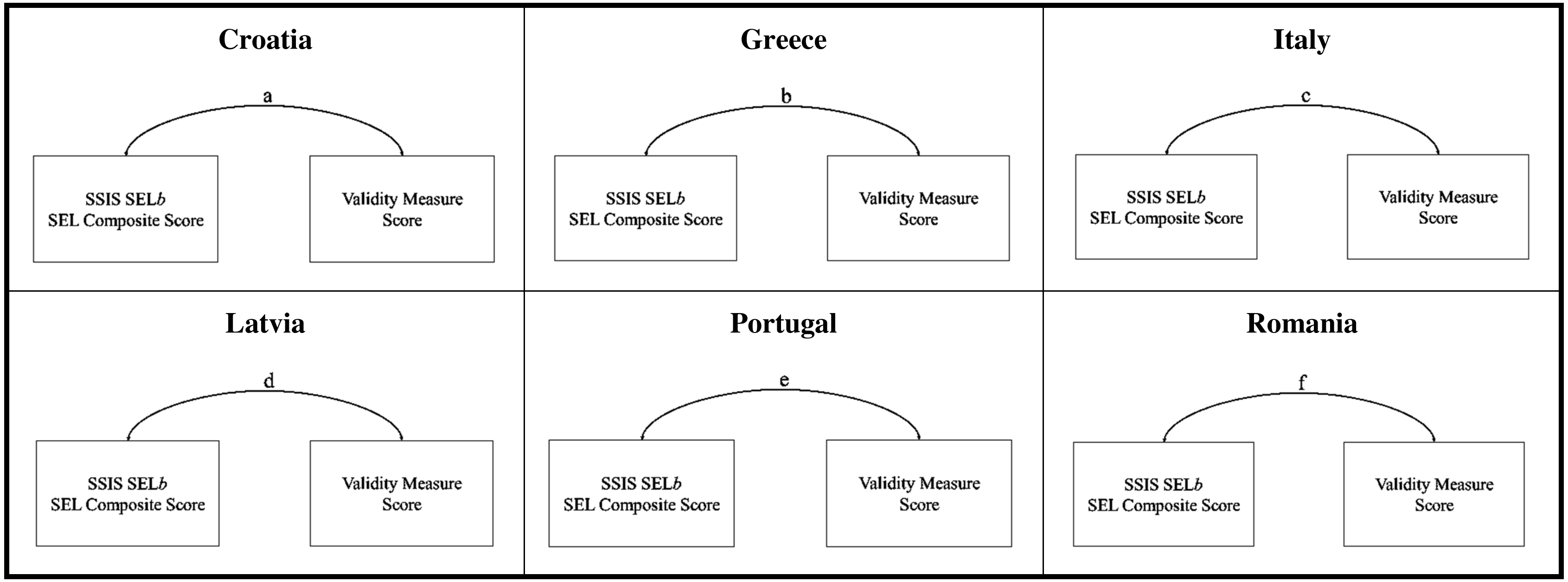

Note. All variables were standardized prior to analysis to render covariances correlations. Cross-country correlations compared by constraining labelled covariances to equality across countries.

References

Achenbach, T. M., & Rescorla, L. (2001). *Manual for the ASEBA (Achenbach System of Empirically-Based Assessment) School-Age Forms and Profiles*. Burlington: University of Vermont, Research Center for Children, Youth, and Families.

Anjos, J. F., Dos Santos, M. J. H., Ribeiro, M. T., & Moreira, S. (2019). Connor-Davidson Resilience Scale: validation study in a Portuguese sample. *BMJ open*, 9(6), e026836. https://doi.org/10.1136/bmjopen-2018-026836

Connor, K. M., & Davidson, J. R. (2003). Development of a new resilience scale: The Connor-Davidson resilience scale (CD-RISC). *Depression and anxiety*, *18*(2), 76-82. https://doi.org/10.1002/da.10113

Costa, P. A., Tasker, F., Ramos, C., & Leal, I. (2020). Psychometric properties of the parent's versions of the SDQ and the PANAS-X in a community sample of Portuguese parents. *Clinical Child Psychology and Psychiatry*, 25(2), 520-532. https://doi.org/10.1177/1359104519891759

Di Riso, D., Salcuni, S., Chessa, D., Raudino, A., Lis, A., & Altoè, G. (2010). The Strengths and Difficulties Questionnaire (SDQ). Early evidence of its reliability and validity in a community sample of Italian children. *Personality and Individual Differences*, 49(6), 570-575. https://doi.org/10.1016/j.paid.2010.05.005

Giannakopoulos, G., Dimitrakaki, C., Papadopoulou, K., Tzavara, C., Kolaitis, G., Ravens-Sieberer, U., & Tountas, Y. (2013). Reliability and validity of the Strengths and Difficulties Questionnaire in Greek adolescents and their parents. *Health*, 2013. https://doi.org/0.4236/health.2013.511239

Giurcă, I. C., Baban, A., Pintea, S., & Macavei, B. (2021). Psychometric properties of the 25-item Connor-Davison Resilience Scale: Preliminary data for a Romanian military population. *Land Forces Academy Review*, 26(1), 31-38. https://doi.org/10.2478/raft-2021-0005

Goodman, R. (1997). The Strengths and Difficulties Questionnaire: A research note. *Journal of child psychology and psychiatry*, *38*(5), 581-586. https://doi.org/10.1111/j.1469-7610.1997.tb01545.x

Grazzani, I., Agliati, A., Cavioni, V., Conte, E., Gandellini, S. Lupica Spagnolo, M., Ornaghi, V., Rossi, F.M.1, Cefai, C., Bartolo, P., Camilleri, O'riordan, M. R. (2022). Adolescents' resilience during COVID-19 pandemic and its mediating role in the association between SEL and mental health. *Frontiers in Psychology,* 153. https://doi.org/10.3389/fpsyg.2022.801761

Lee, E. J., Keyes, K., Bitfoi, A., Mihova, Z., Pez, O., Yoon, E., & Masfety, V. K. (2014). Mental health disparities between Roma and non-Roma children in Romania and Bulgaria. *BMC psychiatry*, 14(1), 1-7. https://doi.org/10.1186/s12888-014-0297-5

Martinsone, B.,, Supe, I. , Stokenberga, I., Damberga, I.., Cefai, C., Camilleri, L., O'Riordan, M., Grazzani. I. (2022). Social emotional competence, learning outcomes, emotional and behavioral difficulties of preschool children: Parent and teacher evaluations. *Frontiers in Psychology*. https://doi.org/10.3389/fpsyg.2021.760782/full

Mikuš, M., Škegro, B., Karadjole, V. S., Lešin, J., Banović, V., Herman, M., ... & Vujić, G. (2020). Maternity blues among creation mothers: A Single center study. *Psychiatria Danubina*, 33(3):342-346. https://doi.org/10.24869/psyd.2021.342

Rodrigues, S., Barbosa-Ducharne, M., Del Valle, J. F., & Campos, J. (2019). Psychological adjustment of adolescents in residential care: Comparative analysis of youth self-report/strengths and difficulties questionnaire. *Child and Adolescent Social Work Journal*, 36(3), 247-258. https://doi.org/10.1007/s10560-019-00614-x

Sharratt, K., Foca, L., Gavriluta, C., Jones, A., & Asiminei, R. (2014). Dimensionality and construct validity of the Romanian self-report strengths and difficulties questionnaire (SDQ). *Romanian Journal of Applied Psychology*, 16(2), 33-39.

Tatalović Vorkapić, S., Slaviček, M., & Vlah, N. (2017). Strengths and difficulties in Croatian preschool children: Validation of the Strengths and Difficulties Questionnaire. *Hrvatska Revija Za Rehabilitacijska Istraživanja*, 53(Supplement), 231-243. https://doi.org/10.1037/t70468-000

Tobia, V., & Marzocchi, G. M. (2018). The strengths and difficulties questionnaire-parents for Italian school-aged children: psychometric properties and norms. *Child Psychiatry & Human Development*, 49(1), 1-8. https://doi.org/10.1007/s10578-017-0723-2

Tsigkaropoulou, E., Douzenis, A., Tsitas, N., Ferentinos, P., Liappas, I., & Michopoulos, I. (2018). Greek version of the Connor-Davidson resilience scale: Psychometric properties in a sample of 546 subjects. *in vivo*, 32(6), 1629-1634. https://doi.org/10.21873/invivo.11424
